# The prevalence and consequences of abdominal rectus muscle diastasis among Finnish women: an epidemiological cohort study

**DOI:** 10.1007/s10029-021-02484-8

**Published:** 2021-08-25

**Authors:** R. Tuominen, T. Jahkola, T. Saisto, J. Arokoski, J. Vironen

**Affiliations:** 1 Department of Plastic Surgery, Helsinki University Hospital and University of Helsinki, PL 266, 00029 HUS Helsinki, Finland; 2 Abdominal Center, Helsinki University Hospital and University of Helsinki, Helsinki, Finland; 3 Department of Obstetrics and Gynecology, Helsinki University Hospital and University of Helsinki, Helsinki, Finland; 4 Department of Physical and Rehabilitation Medicine, Helsinki University Hospital and University of Helsinki, Helsinki, Finland

**Keywords:** Abdominal rectus diastasis, Normative values, ARD classification

## Abstract

**Purpose:**

Post-pregnancy abdominal rectus diastasis (ARD) has raised attention in the field of surgery in recent years, but there is no consensus about when to consider surgery. Our aim was to find out what is the normal inter-rectus distance in fertile aged, female population in Finland and to examine whether there is a linea alba width that would predispose to diastasis-related problems after pregnancy.

**Methods:**

For this prospective cohort study, women participating early pregnancy ultrasound in Helsinki University Hospital Department of Obstetrics and Gynecology during 1.1.2018–8.3.2019, were recruited. The width of linea alba was measured by ultrasound during the early pregnancy ultrasound. Symptoms were measured by questionnaire including Health-Related Quality of Life (RAND-36) and Oswestry Disability Index for back symptoms and disability.

**Results:**

Linea alba width was measured in total of 933 women. The average inter-rectus distance (IRD) among nulliparous women was 1.81 ± 0.72 cm. After one previous pregnancy, the average linea alba width was 2.36 cm ± 0.83 cm and after more pregnancies 2.55 ± 1.09 cm. There was a positive correlation between previous pregnancies and the increased linea alba width (*p* = 0.00004). We did not perceive any threshold value of linea alba width that would predispose to back pain or movement control problems in this cohort, in which severe diastasis (over 5 cm) was rare.

**Conclusion:**

Mean inter-rectus distance in parous population exceeds stated normative values. Moderate ARD (3.0–5.0 cm) alone does not seem to explain low back pain or functional disability in population level. Severe post-pregnancy diastasis (over 5.0 cm) is rare.

## Introduction

Linea alba is the fusion of aponeuroses of the external abdominal oblique, internal abdominal oblique and transversus abdominis muscles and consists of a three-dimensional, structured meshwork of collagen fibers [1]. Anterior abdominal wall biomechanically influences the movements and stability of the lumbar spine [2–4]. It has been suggested that abdominal rectus diastasis (ARD) contributes to back pain [5, 6]. On the other hand, a recent systematic review concluded that there is no significant association between the presence of ARD and lumbo-pelvic pain or incontinence [7]. Another review concluded that antepartum activity level may have a protective effect on ARD and exercise may improve post-partum symptoms of ARD [8].

There are a few publications of inter-rectus distance (IRD) in nulliparous women, Table 1. Beer et al. [9] examined 150 women between 20 and 45 years of age and with a body mass index below 30 kg/m^2^ and the authors concluded IRD up to 2.2 cm being normal measured 3 cm above umbilicus. In porous population, the values were mostly collected relatively soon, 6 months, after pregnancy. Mota et al. [10] examined the width of linea alba in 84 post-pregnancy primipara women showing that in primiparous normative inter-rectus distance is wider than in nulliparous women: at the location 2 cm above umbilicus 1.7–2.8 cm. Mota study also showed that linea alba is widest a few centimeters above umbilicus. Based on Ranney et al. [11] separation of 2.0–3.0 cm between the rectus muscles is considered mild diastasis, 3.0–5.0 cm moderate diastasis and more than 5.0 cm severe. According to literature, the acquired ARD persists approximately in one-third of women after pregnancy [12, 13].

**Table 1:** Available data of normative values of inter recti diameter

Author	Journal	Language	Year	*N*		Measuring point with respect to umbilicus	Measuring timing with respect to giving birth	IRD nulliparous cm	IRD parous, cm
Beer [9]	Clin Anat	Eng	2009	150	Us	3 cm above	Nulliparous	1.3 ± 0.7	
Mota P [10]	*Musculoskelet Sci Pract*	Eng	2018	84	Us	2 cm above	6 months post partum		1.7–2.8
Rath [26]	Sur Radiol Anat	Eng	1996	80	CT, autopsy	Above	cadaver autopsy, CT	1.0	1.0
Rett [27]	Revista Brasileira de Fisioterapia	Por	2009	467	palpation	4.5 cm above and below	Immedately after giving birth		2.7 ± 0.12 primipara, 2.8 ± 0.12 multipara
Mota [28]	Man Ther	Eng	2015	84	Us	2 cm below	6 months post partum		1.53 ± 0.84
Coldron [24]	Man Ther	Eng	2008	184*	Us	above	12 months *n* = 26 *6 months *n* = 39	1.12 ± 0.36	2.07 ± 0.73 (6 months: 2.33 ± 0.84)
Liaw [23]	J Orthop Sports Phys Ther	Eng	2011	60**	Us	2.5 cm above	6 months post-partum	0.85 ± 0.26	1.80 ± 0.72
Turan [29]	Ginekol Pol	Eng	2011	95***	Palpation	3–4 cm above	Over 6 Months	0.15 ± 0.4	0.98 ± 0.35 (primipara) 2.35 ± 1.01 (multipara)

Eng., English; por, Portugal; us, ultrasound; IRD, inter rectus measurement; cm, centimeter

*Coldron study 65 participants were measured at time point 6–12 months post-partum out of 184 participants altogether. There were 26 participants measured at time point 12 months post-partum and 39 participants at 6 months

**Nulliparous (*n* = 20), parous (*n* = 40)

***Nulliparous (*n* = 19), primiparous (*n* = 39) and multiparous (two births) (*n* = 37)

Recently, a working group of the German Hernia Society and the International Endohernia Society presented a proposal of classification of ARD based on the diastasis level (sub-xiphoidal, epigastric, umbilical, infraumbilical, and suprapubic) and the width classification suggested by Ranney [14]. The classification is established particularly to enable precise description of patients being operated for ARD. The proposed classification also takes into consideration other features in the abdominal wall such as concomitant hernias as well as parameters of previous pregnancies, and pain.

There is an increasing awareness of post-pregnancy ARD not only among medical professionals, but also among public, and an increasing number of women who have given birth recently are seeking surgical help for their symptoms that are presumed to be caused by wide IRD. Active physiotherapy is always the primary intervention [7]. There is no consensus on whether and when ARD is a condition requiring operative treatment [15]. Our aim was to find out the normal width of linea alba in normal weight women in Finnish population and study the effects of IRD to back pain, disability and Health-Related Quality of Life (HRQOL). For the background, we searched PubMed for articles using terms “abdominal rectus diastasis” OR” diastasis abdominis recti” OR “linea alba” AND “cohort study” OR “reference values” AND pregnancy.

## Patients and methods

### Design and participants

The study was performed in Helsinki University Hospital, Department of Obstetrics and Gynecology during 1.1.2018–8.3.2019. The width of linea alba was measured by abdominal ultrasound during the early pregnancy ultrasound examination that is offered in public health care in Finland at gestational week 10–13. Each participant received study information and completed a written consent. The study was approved by the Regional Ethics Review Board in Helsinki HUS/3331/2017.

**Fig. 1:**
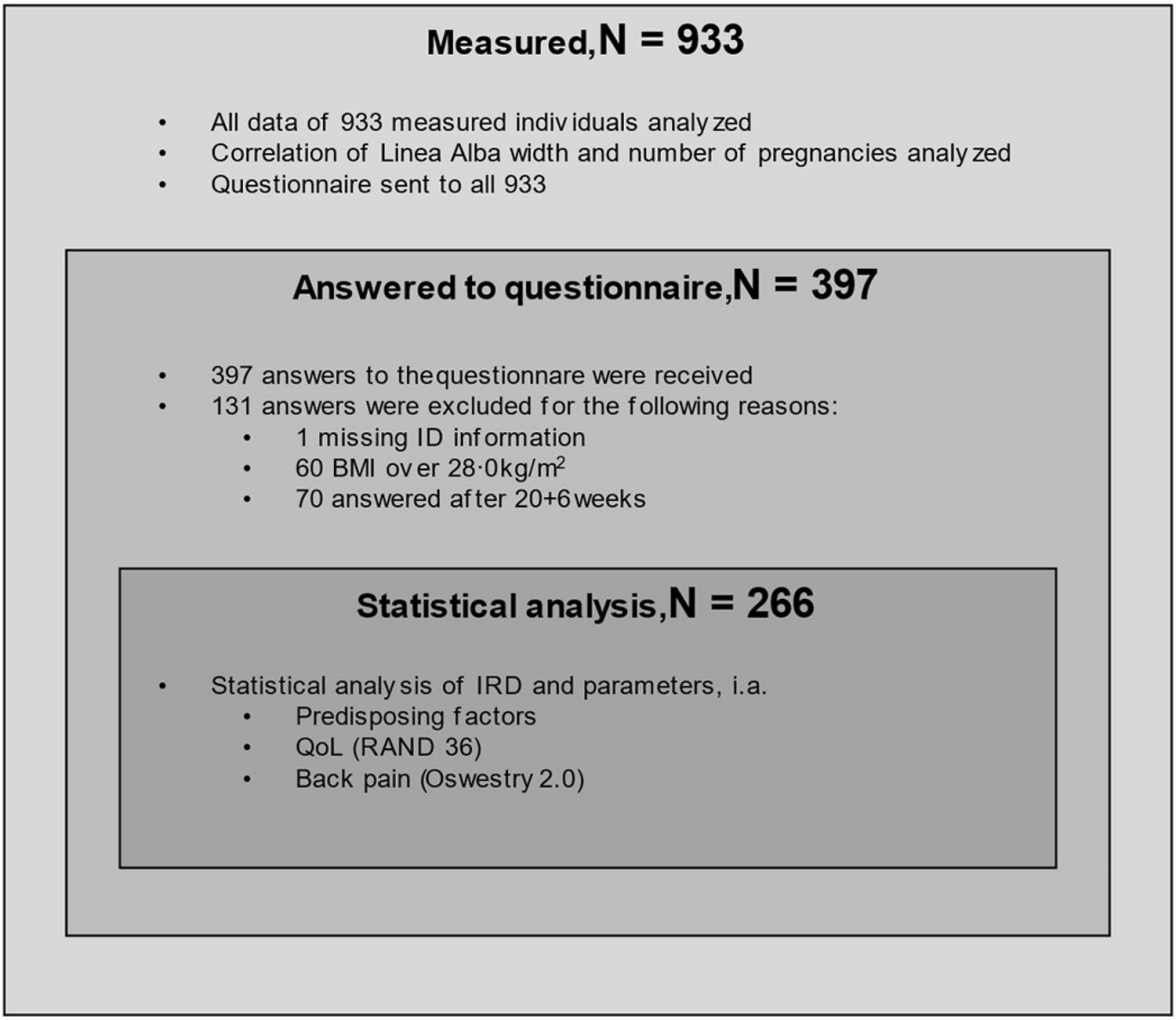
Flowchart of the study. *N*, number; ID, identity; BMI, body mass index; IRD, inter-rectus distance; QoL, quality of life

The measurement in this index pregnancy reflects the effects of previous pregnancies if existing or the nulliparous situation of those individuals who were pregnant for the first time. Flowchart is shown in Fig. 1. Due to practical reasons, every individual during the study period has not been included in the study as these measurements were not performed on very busy days. The exclusion criteria were inability to understand spoken and written Finnish or Swedish, and body mass index (BMI) over 28 kg/m^2^. With obesity, especially with abdominal obesity and substantial amount of visceral fat the stretching of the abdominal wall including linea alba is seen. In our unit, obesity is a contraindication for operative treatment of isolated ARD and we only operate on BMI under 28 kg/m^2^. We chose to concentrate on this group also in this study.

**Fig. 2:**
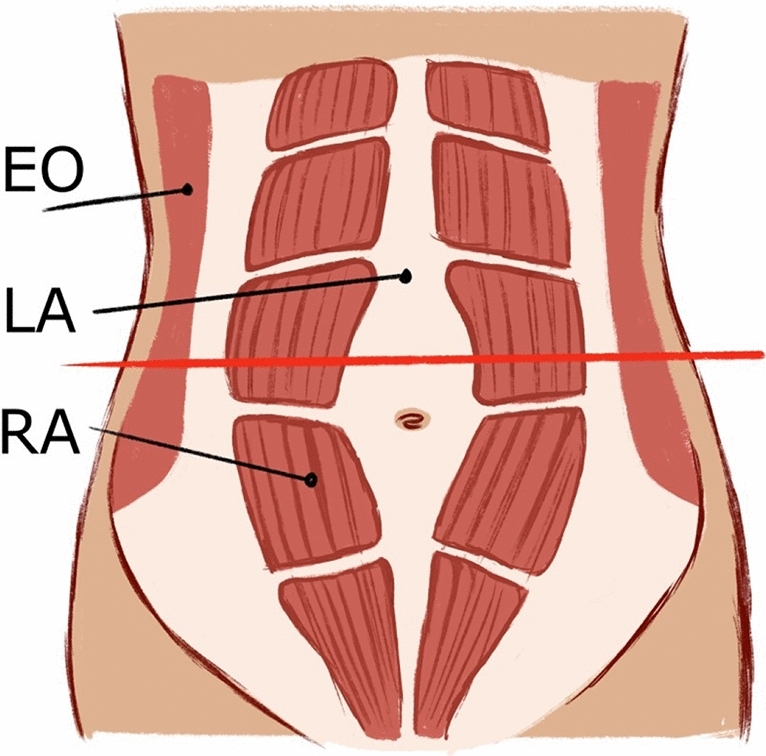
EO, external oblique muscle; LA, linea alba; RA, rectus abdominis muscle. This picture depicts a typical abdominal rectus diastasis. Red line indicates the line of measurement 3 cm above the umbilicus

Twenty midwives contributed to the study and measured the width of the linea alba in women who they estimated to be normal weight. The width was evaluated in supine position, with the neck slightly flexed and with relaxed rectus muscles and normal breathing. The measurement was taken 3 cm above umbilicus on a high-end ultrasound machine, using a high-resolution linear array transducer. The line of measurement is depict in Fig. 2. The focus and depth were adjusted as usual. One representative measurement was taken. All the data were analyzed afterwards, including BMI calculation. The data evaluation revealed that 60 individuals exceeded BMI 28 kg/m^2^ and these participants were excluded from the symptom evaluation.

### Questionnaires

Participants filled an electronical questionnaire sent to a given email including RAND36 validated Quality of Life Questionnaire [16] and Oswestry 2.0 low back disability index [17]. In RAND36 physical functioning, pain and general health perceptions of oneself were analyzed. In addition to previously described established questionnaires also other variables were inquired such as weight and height, details of previous pregnancies (the number of previous pregnancies, cesarian sections and vaginal births, the sizes of previous babies, own maximum weight gain), urinary symptoms (the amount of sanitary pads used because of incontinence, the symptoms of incontinence with mild and also with moderate physical stress and the social burden caused by incontinence), sport activity (the times one exercises in a way getting out of breath or sweat), and the satisfaction of the contour of the abdominal wall, Table 2.

**Table 2:** Parameters of questionary and the scoring algorithm

Question number		Unit of measure						
1	Weight before pregnancies	kg						
2	Maximum own weight in any pregnancy*	kg						
3	Present weight	kg						
4	Number of vaginal births	n						
5	Number of Cesarian sections	n						
6	Number of twin pregnancies	n						
7	Number of premature births	n						
8	Maximum weight of a baby	kg						
9	How many times you exercise in a way you get out of breath or sweat?		Daily = 1p	4–6 times a week = 2p	2–3 times a month = 3p	Once a week = 4p	2–3 times a month = 5p	Once or less in a year = 6p
10	How often does urine leak when you are physically notably active (like), running or sneezing		Never = 0p	Seldom = 1p	Often = 2p			
11	How often does urine leak when you are physically mildly active (i.e. walking or standing up)		Never = 0p	Seldom = 1p	Often = 2p			
12	Overall, how much does leaking urine interfere with your life?		Not at all = 0p	Sometimes = 1p	Often = 2			
13	Select the number of protective garments for urine leakage you use per day		None = 0p	One = 1p	Two or more = 2p			
	Incontinence score = Questions 10–13 points summed							
12	Are you satisfied with the contour of your abdomen		Strongly agree = 1p	Agree = 2p	Neither agree nor disagree = 3p	Disagree = 4p	Strongly disagree = 5p	

The electronic questionnaire enabled answering at any time point chosen by participant. The median answering time point was 15 ± 2.1 gestational week but some participants postponed their answers several weeks. Those answering after week 20 + 6 were excluded as it is known that after gestational week 24 lower back pain is more common [18].

### Statistical methods

All statistical analyses were made using NCSS 12 Statistical Software. The alpha level for all statistical tests was set to 0.05. Equal-variance *t* test was used to compare numerical variables when distributions were approximately normal. Aspin-Welch unequal-variance *t* test was also utilized. Mann–Whitney *U* test was used when the variable distributions were non-normal. The Pearson Chi-Square test was used to assess the linea alba width and previous cesarean sections, and pregnancies. The correlation between linea alba width and back pain was analyzed with Pearson linear correlation test.

## Results

Altogether 933 women were examined for the study and 397 answered the questionnaire. Of them, 266 participants met the inclusion criteria for symptom evaluation. The median for answering was 15 ± 2.1 gestational week. The flowchart of the study is shown in Fig. 1.

**Fig. 3:**
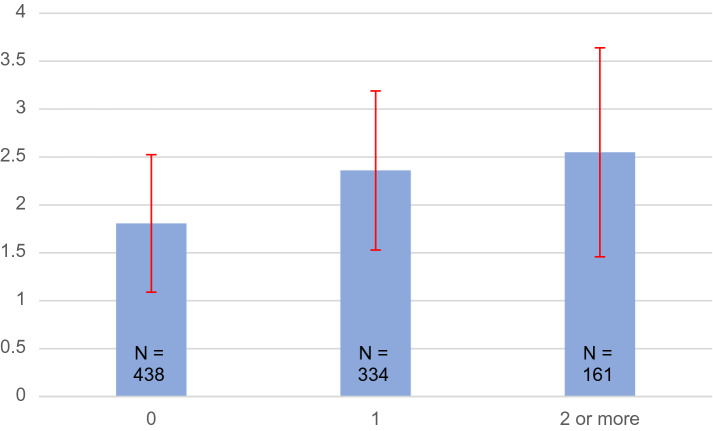
Inter-Rectus Distance as a function of given births

**Fig. 4:**
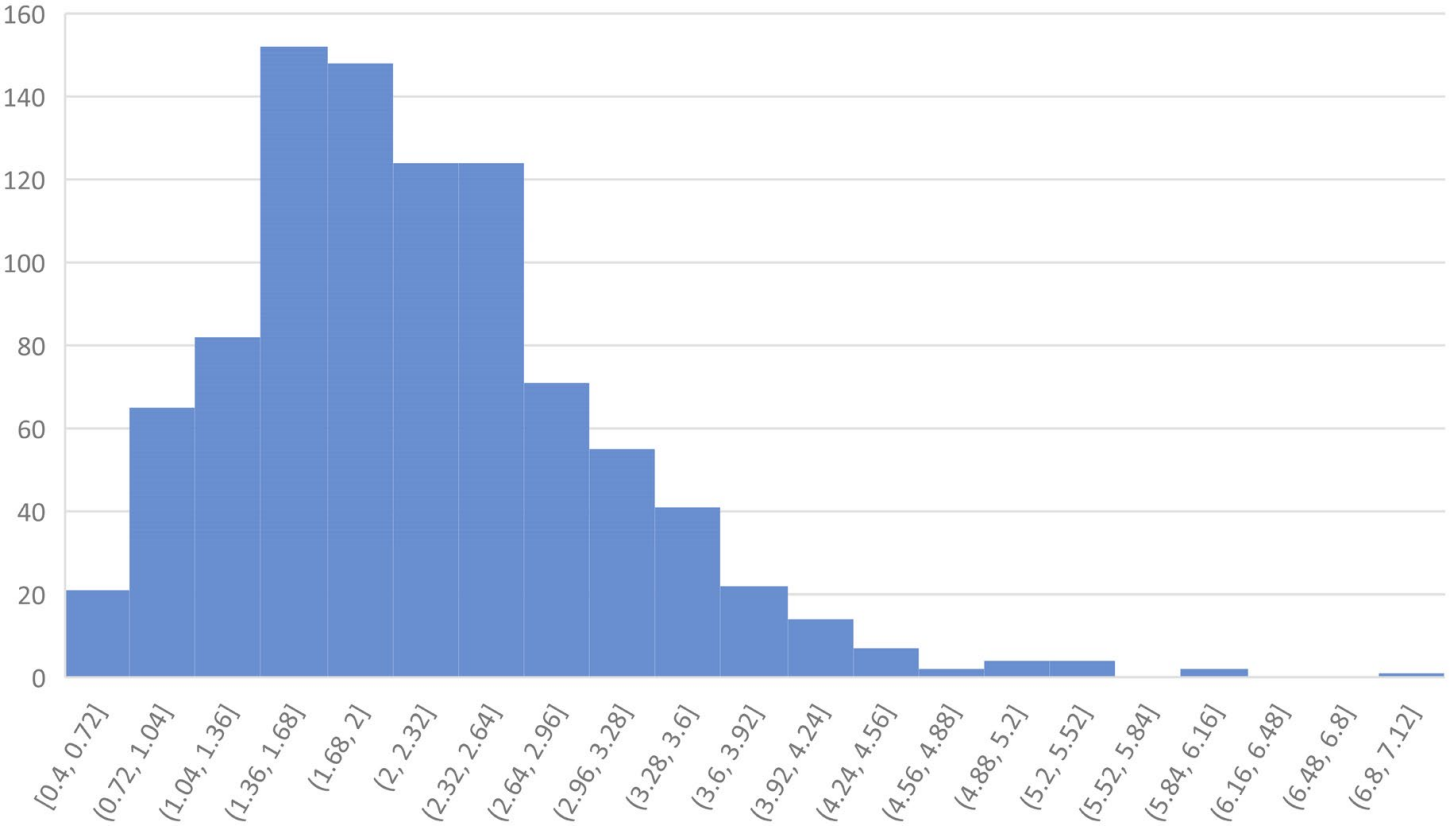
Histogram of the Inter-rectus distance observations

In the complete data of 933 measurements, also containing those individuals who did not answer the questionnaire the mean linea alba width among nulliparous women was 1.81 ± 0.72 cm. After one previous pregnancy, the average linea alba width was 2.36 ± 0.83 cm and after more pregnancies 2.55 ± 1.09 cm. There was a positive correlation between the number of previous pregnancies and the increased linea alba width (*p* = 0.00004), Fig. 3. The range of linea alba width in this Finnish female fertile aged population was 0.4–7.0 cm, Fig. 4.

The background variables and potential risk factors were compared with diastasis defined as IRD below 3.0 cm and IRD ≥ 3.0, Table 3. The value 3.0 cm was chosen as it is the upper limit for mild diastasis according to Ranney. IRD did not have a statistical correlation to BMI (at the time of answering the questionnaire), exercise training customs, age, weight change during the present pregnancy, weight change in previous pregnancies, maximum newborn or infant weight in previous births, or previous cesarean sections.

**Table 3:** Background variables and potential risk factors for diastasis with groups of Ranney mild (< 3 cm) and moderate/severe (≥ 3 cm) abdominal rectus diastasis

	*n*, linea alba < 3 cm	Mean	*n*, linea alba ≥ 3 cm	Mean	Test T-Statistic		*p*
Age (years)	266	31.5	37	32.2	Equal-Variance T-Test		0.39
BMI at the time of answering (kg/m^2^)	266	22.9	37	23.5	Equal-Variance T-Test		0.16
Weight change during early pregnancy in present pregnancy, kg	266	2.77	37	3.60	Equal-Variance T-Test		0.17
Regular exercise training	265	3.12	37	3.16	Equal-Variance T-Test		0.85
Weight change in previous pregnancies, kg	127	13.43	33	13.76	Equal-Variance T-Test		0.77
Baby weight in previous pregnancy, g	106	3472.5	29	3587.9	Equal-Variance T-Test		0.24
Number of previous pregnancies 0/1/2 or more		139/91/36		5/25/7	Pearson's Chi-Square		0.00004
Cesarian sections in previous pregnancies, *n*	16		2		Pearson's Chi-Square		0.88

The only correlation was the number of previous pregnancies with the wider IRD

g, gram; *n*, number; BMI, body mass index

**Fig. 5:**
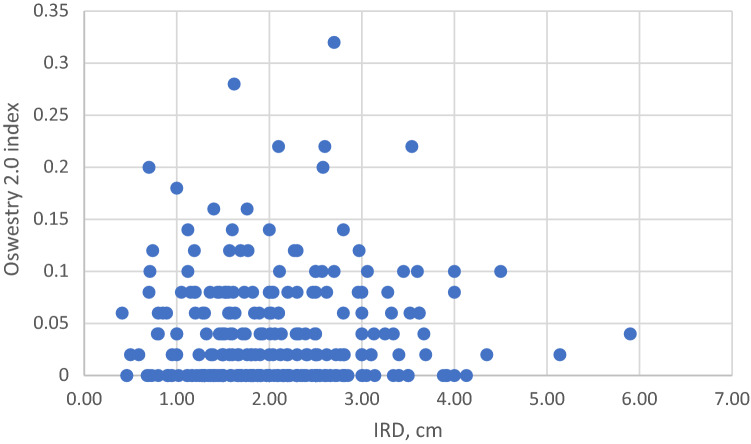
Correlation of Oswestry Index and IRD. *cm* centimeter, *IRD* inter-rectus distance

**Fig. 6:**
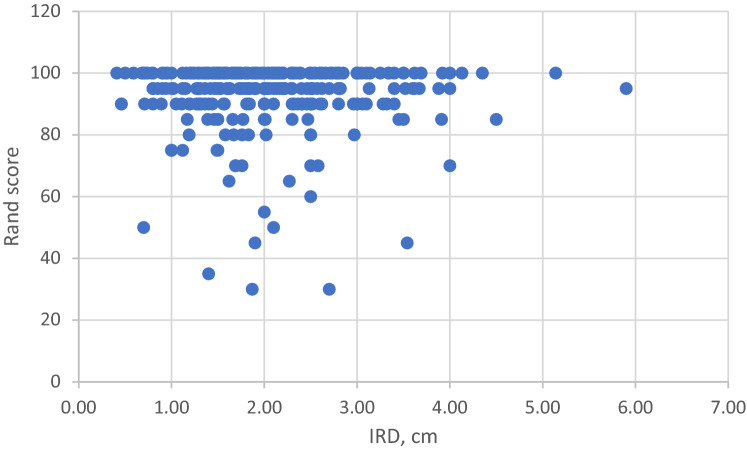
Correlation of RAND36 domain of Physical Functioning and IRD. *cm* centimeter, *IRD* inter-rectus distance

It was not possible to outline a threshold value of IRD that would predispose to disability. Figure 5 shows Oswestry Disability Index and Fig. 6 a RAND36 domain of Physical Functioning as a function of IRD; there is no correlation in either of them. The data were analyzed with a cut off value of 3.0 cm. No differences between groups IRD < 3.0 cm and IRD ≥ 3.0 in RAND36 Quality of Life Index nor in the RAND Domains of Physical functioning, Bodily pain, General health, or psychological health was seen. Nor were there differences in Oswestry Back Pain Index or Oswestry topics on Standing, Lifting or Pain intensity, Table 4. There was a statistical correlation between IRD and incontinence and the satisfaction on the esthetics of one´s abdominal wall. Incontinence scale in groups W1 (IRD < 3.0 cm) and W2 (≥ 3.0) was 0.39 and 0.86, respectively (*p* = 0.011). Satisfaction to abdominal contour with a Likert scale from 1 to 5 (with 0 being totally satisfied and 5 not satisfied at all) was 2.23 in W1 and 2.86 in W2 (*p* = 0.04).

**Table 4:** Statistical analyses of variables of W1 (IRD < 3.0 cm) and W2 (≥ 3.0) groups

Scale	Group	*N*	Mean	Median	*p*	Reference
Quality of life (RAND36) domain: physical functioning	W1	263	93,1	95		93.3*
	W2	37	93,5	95	0.97	
Quality of life (RAND36) domain: bodily pain	W1	261	84,9	90		80.5*
	W2	37	86,6	90	0.28	
Quality of life (RAND36) domain: general health	W1	261	77.3	80		74.9*
	W2	37	76.1	75	0.28	
Quality of life (RAND36) domain: physical role functioning	W1	262	82.7	100		83.5*
	W2	37	89.2	100	0.12	
Oswestry Low Back Pain Questionnaire: total index	W1	262	0.035	0.02		
	W2	37	0.038	0.02	0.33	
Oswestry Low Back Pain Questionnaire: topic lifting	W1	262	0.19	0		
	W2	37	0.13	0	0.48	
Oswestry Low Back Pain Questionnaire: topic standing	W1	262	0.37	0		
	W2	37	0.4	0	0.66	
Oswestry Low Back Pain Questionnaire: topic pain intensity	W1	262	0.28	0		
	W2	37	0.29	0	0.73	
Incontinence	W1	266	0.39	0		
	W2	37	0.86	0	0.011	
Satisfaction to abdominal countour	W1	265	2.23	2		
	W2	37	2.86	3	0.04	

*Finnish RAND-36 normative values according to Aalto et al. 1999 188 (female aged between 25–34 years)

## Discussion

Abdominal rectus diastasis is a raising topic in the field of abdominal wall defects and treatment [19]. It is not clear where to draw the line between normal anatomy and ARD diagnosis and whether there is an IRD width that would predispose to ARD-related problems [15]. As more patients with post-pregnancy diastasis have been referred to Helsinki University Hospital Department of Plastic Surgery and Abdominal Center, there was a need to study what can be considered normal and how does ARD affect in population level.

We conducted our study in the connection of first trimester ultrasound examination as that way it was possible to reach a large cohort of women and measure the linea alba width at the same time as pregnancy scanning was done. In a systematic review, ultrasound was considered an adequate method to assess linea alba width [20]. Interrater error has been shown to be acceptable [21]. In previous studies, measurement error was the greatest at the superior border of the umbilicus. Below the umbilicus measuring can be more challenging due to a loss of posterior rectus sheath definition and greater difficulty in visualizing the rectus sheath at this level [21]. We chose measuring point 3 cm above umbilicus as it is in line with previous studies and to ensure the lowest error with multiple rater setup. Midwives who performed the measurements are professionals with ultrasound as they use it daily for pregnancy follow up.

The Ranney classification suggests IRD above 2.0 cm to be considered mild ARD [11]. In our study, the average width of linea alba in nulliparous population was 1.81 ± 0.72 cm that is in line with Ranney classification and also similar to Beer classification of up to 2.2 cm being normal. In the present study, all participants were pregnant which can affect the quality of linea alba fascia. However, the fact that in nulliparous population the mean IRD was in line with previous studies supports our assumption that the IRD does not change significantly during the first trimester. At gestational week 13, the fetus is 6 cm long and the size of the uterus is approximately the size of a grapefruit [22] so the mechanical stretching force to the abdominal wall is small.

Most of the previous epidemiological studies discussing parous cohorts have been performed immediately or only six months after pregnancy. The longitudinal study of Sperstand et al. [13] suggests that IRD’s decreasing behavior continues further after 6 months. Deductively, the values stated in previous studies may well be above the correct ones as the data is collected early after pregnancy. In previous studies, in which the IRD was measured from approximately same horizontal level that we used in this study (3 cm above umbilicus), the parous normal values were 1.7–2.8 in Mota series [10] and 1.80 ± 0.72 cm according to Liaw [23]. In Coldron study [24], the exact anatomical level was not specified, but the mean IRD was 2.07 ± 0.73. In our data already after one single pregnancy, the mean IRD was 2.36 cm ± 0.83 cm, and after more pregnancies 2.55 ± 1.09 cm which is more than in previous studies. This means that in our relatively large data of 495 measured IRDs in parous population mild diastasis according to Ranney classification is common. Due to incongruities of our and previous data with parous population more studies are needed to address normative IRD values after 12 months or more after pregnancy. If future studies reveal that most of parous individuals have IRD large enough to set ARD diagnosis, the upper limit to normal IRD needs to be re-evaluated.

In literature where the predisposing factors and effects of diastasis have been studied, the definition of ARD has varied in a wide scale—as low as 16 mm has been considered ARD. Akram concluded also that antepartum activity level may have a protective effect on RD and exercise may improve post-partum symptoms of RD [8]. In our data, we did not find any correlation of IRD and sport activity. We did not find any correlation with disability or quality of life and IRD with the cut of point of 3.0 cm. Our data suggest that moderate diastasis does not differ from normative and mild IRD in population level. One possible explanation is that ARD predisposes to problems only when it is severe. In this study, there were only two participants with BMI under 28 having severe diastasis over 5 cm. Sperstad et al. [13] used the principle of four or more fingerbreadths implicating severe diastasis. Though the precision of these results might be questioned, their finding of only 2 moderate and none out of 178 severe ARD is in line with our study. Future studies will hopefully address the question whether severe ARD is an indication for operative treatment. In the recent surgical studies of symptomatic ARD, the average IRD has been severe or close to severe. In RCT by Emanuelsen [6], the average operated IRD was 4.0 cm and in our retrospective operative treatment study of symptomatic ARD with PSUM-method 5.2 cm [25]. In our experience, women with normal weight and wide diastasis suffer from the symptoms most. In connection with obesity, widened IRD is natural to allow space for visceral fat. We feel in such situations ARD should not be operated as with doing so the intra-abdominal pressure might rise excessively. Further in connection of obesity the anterior abdominal wall is often firm and not loose, and the effects and indications of surgery would probably differ from normal weight patients.

The increased demand to operative treatment necessitates more studies to recognize those individuals who are most likely to benefit of invasive treatment.

According to this study, mild and moderate diastasis alone does not seem to predispose to diastasis-related difficulties and, therefore, these conditions alone are not an indication to operate. The upper limit to IRD that is to be considered normal might be higher than stated so far.

## Conclusion

Mild and moderate diastasis alone does not seem to play an important role in disability and back pain.

Further studies are needed before assessment of the effects of severe ARD can be made. The rarity of severe diastasis necessitates large cohorts in the future studies.
